# Differences in Behavioral Inhibitory Control in Response to Angry and Happy Emotions Among College Students With and Without Suicidal Ideation: An ERP Study

**DOI:** 10.3389/fpsyg.2020.02191

**Published:** 2020-09-01

**Authors:** Lin Lin, Chenxu Wang, Juanchan Mo, Yu Liu, Ting Liu, Yunpeng Jiang, Xuejun Bai, Xia Wu

**Affiliations:** ^1^Key Research Base of Humanities and Social Sciences of the Ministry of Education, Academy of Psychology and Behavior, Tianjin Normal University, Tianjin, China; ^2^Faculty of Psychology, Tianjin Normal University, Tianjin, China; ^3^Center of Collaborative Innovation for Assessment and Promotion of Mental Health, Tianjin, China

**Keywords:** behavioral inhibitory control, ERPs, two-choice oddball paradigm, impulsivity, suicidal ideation

## Abstract

Suicidal ideation is one of the strongest predictors of suicide. A large number of studies have illustrated the important effect of impulsivity on suicidal ideation, and behavioral inhibitory control (BIC) is a specific manifestation of impulsivity. The goal of the present study is to evaluate the difference in BIC in response to happy and angry emotions between individuals with or without suicidal ideation to reveal the underlying mechanism of the effect of impulsivity on suicidal ideation when accounting for the effect of emotion. Combining the ERP technique and the two-choice oddball paradigm, a total of 70 college students were recruited to participate in this study. The Beck Scale for Suicidal Ideation–Chinese Version was used to identify whether the participants had suicidal ideation. There were 30 participants in the risky-suicidal ideation (SI) group and 19 participants in the non-suicidal ideation (NSI) group. The results showed that the reaction time of the SI group was longer than that of the NSI group for happy emotions. At the electrophysiological level, the P3 amplitude of the NSI group was larger than that of the SI group regardless of the electrode sites and valence, and the P3 component elicited by angry faces was larger than those elicited by happy faces in the SI group. These findings suggest that individuals without suicidal ideation have better BIC, and the SI group has more difficulty controlling their responses to happy emotions than their responses to angry emotions.

## Introduction

Suicidality is becoming a serious threat to the health of college students and has become the second leading cause of death among college students in recent years (Heron, 2017). Suicidal ideation (SI) refers to individuals who currently have plans and wishes to die by suicide but have not initiated any overt suicide attempt (Beck et al., 1979), which is a main factor in the psychological process leading to suicidal behavior (Deykin and Buka, 1994). Impulsivity is a noteworthy personality trait that is associated with SI and has an important effect on SI (Klonsky and May, 2010). A number of studies have illustrated that the risk of suicidal ideation increases with the level of impulsivity (Auerbach et al., 2016; Wang et al., 2019). Thus, to take effective measures to protect susceptible individuals from emerging SI, it is essential to clarify the underlying mechanism by which impulsivity influences SI.

As a personality trait, impulsivity is conceptualized as “a predisposition toward rapid, unplanned reactions to internal or external stimuli without regard to the negative consequences of these reactions” (Moeller et al., 2001). Barratt and Patton (1983) developed the Barratt Impulsiveness Scale to measure impulsivity. They theorized that impulsivity has three facets: attentional impulsivity (AI) is characterized by difficulty concentrating; non-planning impulsivity (NPI) is characterized by a lack of impulse control and a lack of planning for the future; motor impulsivity (MI) is characterized by acting without thinking. In addition to the self-report scale, a variety of tasks have been developed to assess impulsivity more objectively, such as the go/no-go task and the two-choice oddball paradigm. In particular, the go/no-go task consists of the presentation of a continuous series of “go” (i.e., the target) cues, to which participants are required to respond as accurately and quickly as possible, and “no-go” cues, which require participants to inhibit motor responses. However, motor responses are involved in the task, so the effects of inhibitory control observed in the task are likely to be contaminated by response-related processes. Therefore, the well-established two-choice oddball paradigm improves upon the aforementioned shortcomings of the go/no-go task and reflects one of the most important components of impulsivity, i.e., the ability to suppress inappropriate actions and thoughts, which can be measured by behavioral inhibitory control (BIC) (Logan et al., 1997; Yuan et al., 2017). Participants are required to respond to both standard (70%) and deviant (30%) stimuli by pressing different keys as quickly as possible (rather than one response to the “go” stimuli in a go/no-go task). The responses to standard stimuli would be a dominant response, which needs to be suppressed to ensure a correct response to the deviant stimuli. Thus, the two-choice oddball paradigm reflects the effect of BIC purely by subtraction between deviant and standard stimuli (Yuan et al., 2008a). In light of the aforementioned advantages, the two-choice oddball paradigm is adopted in this study.

Event-related potentials (ERPs) are often measured during the two-choice oddball paradigm to examine the cognitive processes underlying BIC. N2 and P3 components related to BIC have been consistently found across studies. N2 is a negative component that reflects conflict monitoring with the largest amplitude in frontal electrode sites (Yeung et al., 2004; Wang et al., 2011); P3 is a late positive component that reflects the inhibitory process itself with the largest amplitude in parietal electrode sites (Albert et al., 2010; Kamp and Donchin, 2015). Therefore, N2/P3 components of the difference wave (subtraction between deviant and standard stimuli) induced in the two-choice oddball paradigm are an effective index of BIC. BIC is an interrelated mechanism of impulsivity, and it has frequently proven that impulsivity levels increase as BIC decreases (Enticott et al., 2006). A decrease in BIC could predict suicidal risk (Venables et al., 2015). Thus, focusing on the relationship between BIC and SI could reveal the underlying mechanism of the effect of impulsivity on SI. The first goal of the present study is to compare the difference in BIC (indexed by N2 and P3 components) between SI and NSI groups to elucidate the neural correlates of impulsivity and SI.

Furthermore, the complex interactions between human emotional activity and inhibition control have been proven by a handful of studies (Gross, 2007; Rowe et al., 2007). For instance, as a product of out-of-control behavior, aggression is often closely related to negative emotions (Shafritz et al., 2006; Stewart et al., 2010), and the performance of cognition and behavioral control can lead to significantly different emotional experiences of the same event (Gross, 2007). Similarly, there is a significant negative correlation between impulsivity and the emotional stability of individuals (Carver and White, 1994), which suggests that low levels of BIC are pronounced under negative emotional states (Posner et al., 2002). Most of the aforementioned results were obtained from healthy participants. Emotion is closely related to the development of suicidal ideation (Heffer and Willoughby, 2017), and negative emotion is also considered an important risk factor for suicidal ideation (Brausch and Decker, 2014). Emotional vulnerability is a risk factor that has been repeatedly proven to contribute to suicidal ideation, suicidal behavior, and an increased likelihood of future suicide attempts (Arria et al., 2009; Palmierclaus et al., 2012). Therefore, what effect do emotions with different valences have on the BIC of individuals with suicidal ideation? Specifically, the study by Jollant et al. (2008) illustrates that individuals with a history of suicidal attempt have an increased sensitivity to disapproval from others, a higher propensity to act on negative emotions (especially to angry faces), and reduced attention to mildly positive stimuli. Along these lines, the second goal of the present study is to directly compare the BIC of individuals with or without SI under angry emotions and happy emotions to reveal how the two emotions impact the relationship between BIC and SI.

In summary, there are two highlights in the present study. The first is the difference in BIC of individuals with or without SI; the second is the effect of angry and happy emotions on BIC in the two groups. To this end, the study used a two-choice oddball paradigm combined with ERP technology to measure the BIC of individuals accurately. We predicted that there would be significant differences in BIC (indexed by N2 and P3 amplitudes) between participants with or without SI, and we also predicted that the N2 and P3 amplitudes would differ under angry and happy emotions in the two groups.

## Materials and Methods

### Subjects

In total, 799 college students as paid volunteers from Tianjin Normal University participated in the present study. All participants were healthy, right-handed, with normal or corrected to normal vision, and signed an informed consent form before the experiment. To distinguish whether the participants had suicidal ideation, all of them completed twice measurements of the Beck Scale for Suicidal Ideation–Chinese Version (BSI-CV) (Li et al., 2011), taking the 4–5 items as the standard of whether they had suicidal ideation (the total score of two items is greater than or equal to 1 suggest they had suicidal ideation). According to statistics, 139 of them had suicidal ideation in both two measurements, 479 of them had no suicidal ideation in both two measurements, and 181 of them had suicidal ideation in one of the measurements. A total of 70 participants (17 males, 53 females, age range from 18 to 23, mean age = 19.42) from 139 suicidal ideation and 479 non-suicidal ideation participated in the ERP experiments by telephone invitation.

Before the ERP experiment, the invited 70 participants completed the BSI-CV again and the Center for Epidemiological Studies Depression Scale (CES-D; 2010; Zhang et al., 2010). They were classified as suicidal ideation group and non-suicidal ideation group according to the criteria as follows: (1) the participants in risky-suicidal ideation group (SI) scored not equal to 0 in the 4–5 items of BSI-CV (suicidal ideation) and scored not equal to 0 in the 6–19 items of BSI-CV (suicidal risk). A total of 30 participants in the SI group, but 2 of them were excluded because of too many EEG artifacts (artifacts to reject were 86.8% and 92.1%), yielding 28 participants (6 males and 22 females, age range from 18 to 21, mean age = 19.20), entered the statistical analysis. 2) The participants in non-suicidal ideation group (NSI) scored equal to 0 in the 4–5 items of BSI-CV (non-suicidal ideation) and scored equal to 0 in the 6–19 items of BSI-CV (non-suicidal risk). A total of 19 participants in the NSI group, but 1 of them was excluded because of the extreme value of EEG data and 2 of them were excluded because of too many EEG artifacts (artifacts to reject were 92.1 and 87.1%), yielding 16 participants (2 males and 14 females, age range from 18 to 23, mean age = 19.36), entered the statistical analysis. The remaining 21 participants who did not meet the aforementioned grouping criteria were not included in the statistical analysis. The *t*-test for the scores of CES-D showed a significant difference in depression between the two groups (*t* = 3.34, *p* = 0.002). The level of depression in the SI group (42.64 ± 11.02) was significantly higher than that in the NSI group (31.94 ± 8.66), indicating the grouping criteria of this study had a good validity (Bronisch and Wittchen, 1994; Rockett et al., 2007).

### Stimuli

The emotional faces employed in the present study were chosen from the Chinese Facial Affective Picture System (CFAPS; Wang and Luo, 2005). Before the experiment, 36 college students evaluated the identification of emotional type (happy, angry, and neutral), as well as the valence and arousal of the emotional faces. The identification of emotional types of faces (half of male and female) was rate greater than 70% by the students selected for the experiment (e.g., 70% of the students considered that a happy face did express a happy mood). The selected faces included 2 neutral faces as frequent standard stimuli, 12 faces as anger deviant stimuli, and 12 faces as happy deviant stimuli. There was a significant difference in valence between happy and angry faces (*t* = 23.67, *p* < 0.001), and there was no significant difference in arousal (*t* = −1.10, *p* = 0.285). Contrast and luminance levels of the pictures were also controlled. All the pictures were identical in size and resolution (4°8′ × 6°8′, 100 pixels per inch).

### Procedure

**FIGURE 1:**
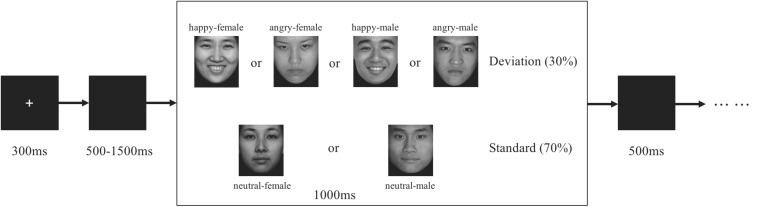
Schematic illustration of the experimental procedure and the stimulus examples. Each trial presented a single deviant or standard stimulus. Participants press the “F” key and “J” key as the response for standard and deviant stimuli, respectively.

Participants were seated in a quiet room at approximately 65 cm from a computer screen. Stimuli were presented using E-Prime version 2.0 (Psychological Software Tools, Pittsburgh, PA, United States). In each trial (see Figure 1), a 300-ms fixation cross was presented, which was followed by a blank screen whose duration varied randomly for 500–1500 ms. Then, a stimulus picture appeared on the screen. Participants were instructed to press the key on the keyboard with their corresponding finger as accurately and quickly as possible. If the standard picture (neutral faces) appeared (70%), press “F” and if the deviant picture (happy or angry faces) appeared (30%), press “J”. The stimulus picture was terminated by a key pressing or was terminated when it elapsed for 1000 ms. Then 500 ms of blank screen ended the trial. The present study consisted of eight trials for practice and participants who achieved 100% accuracy could enter the formal experiment. The formal experiment comprised 12 blocks (half of male and female, and one block presents one single gender), which consisted of 60 trials (18 trials of deviation and 42 trials of standard) with a total of 720 trials. The 1-min break lasted between each block, yielding a total 40 min of the whole experiment.

### ERP Recording and Analysis

Electroencephalography (EEG) was recorded from 64 scalp sites using tin electrodes mounted in an elastic cap (Curry 7 system produced by Neuroscan company), with the references on the left and right mastoids for offline ERP computation (average mastoid reference; Luck, 2005) and a ground electrode on the medial frontal aspect. The vertical electrooculograms (EOGs) were recorded supra- and infra-orbitally at the left eye. The horizontal EOG was recorded from the left versus right orbital rim. The EEG and EOG were amplified using AC recording with the bandpass of 0.05–100 Hz (FIR filter) at a sampling rate of 1000 Hz. Data acquisition was not started until all impedance values were below 5 kΩ.

Signal processing and offline analysis were performed in MATLAB using the EEGLAB toolbox (Delorme and Makeig, 2004) and ERPLAB toolbox (Lopezcalderon and Luck, 2014). All data were re-referenced to the average of the left and right mastoid electrodes and bandpass filtered with low pass 30 Hz (24 dB/oct). Then, ERP waveforms were time-locked to the onset of stimuli and the average epoch was 1000 ms, including a 200-ms pre-stimulus baseline. Artifact detection and rejection were conducted on epoched uncorrected data files to identify and remove trials containing blinks and large eye movements at the time of stimulus presentation (mean EOG voltage exceeding ± 100 μV were excluded). Epochs with large artifacts (exceeding ± 100 μV) were excluded from analysis (Kudinova et al., 2015). Next, ERP data were baseline corrected to the mean amplitude of the pre-stimulus interval. Finally, ERP data for the correct response in each valence condition were overlapped and averaged separately. There were 99.92 trials for angry, 99.79 trials for happy, and 484.68 trials for neutral in SI group; 98.56 trials for angry, 98.50 trials for happy, and 483.69 trials for neutral in NSI group.

**FIGURE 2:**
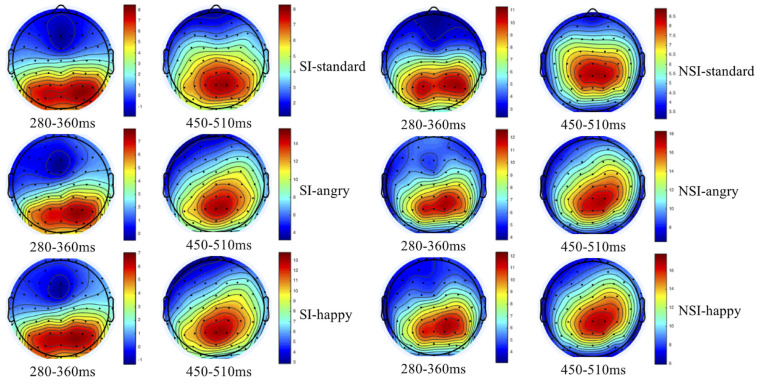
Topographical maps of voltage amplitudes for original waveform at 280–360 ms and 450–510 ms in SI group (left panel) and NSI group (right panel). SI, risky-suicidal ideation; NSI, non-suicidal ideation.

**FIGURE 3:**
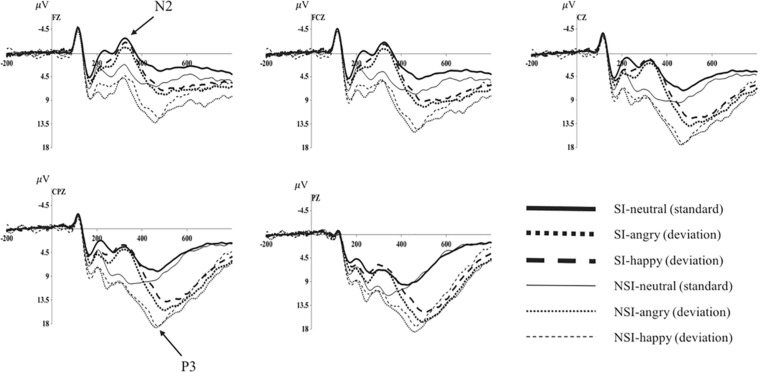
Average of original ERPs at Fz, FCz, Cz, CPz, and Pz for neutral, angry, and happy conditions in SI group and NSI group. SI, risky-suicidal ideation; NSI, non-suicidal ideation.

As shown by the topographical maps and ERP’s grand averaged waveforms, ERP induced by deviant stimuli and standard stimuli were separated from about 150 ms, and the difference continued until about 700 ms. It was mainly composed of N2 (280–360 ms) and P3 (450–510 ms) on the waveform, and these components were largest at frontal and central-parietal sites (see Figures 2, 3). Thus, we selected the following 15 electrode sites for statistical analysis: F1, Fz, F2 (three frontal sites); FC1, FCz, FC2 (three frontal-central sites); C1, Cz, C2 (three central sites); CP1, CPz, CP2 (three central-parietal sites); P1, Pz, P2 (three parietal sites). The mean amplitudes (from stimulus onset to the peak of each component) and the peak latencies were determined with an automated recognition by the ERPLAB toolbox. A three-way repeated measures ANOVA was conducted for the mean amplitude and latency of each component by SPSS 24.0. ANOVA factors were valence condition (two levels: happy and angry) and electrode site (15 sites: frontal sites F1, Fz, F2; frontal-central sites FC1, FCz, FC2; central sites C1, Cz, C2; central-parietal sites CP1, CPz, CP2; and parietal sites P1, Pz, P2) as within-subjects factor, and the group (two levels: risky-suicidal ideation and non-suicidal ideation) as between-subjects factor. The degrees of freedom of the *F*-ratio was corrected according to the Greenhouse–Geisser method. Bonferroni–Holm method was used for *post hoc* comparisons if significant main or interaction effects were found.

## Results

### Behavioral Data

The mean RTs and standard errors of each condition in both groups are presented in Table 1. Errors were rare, all participants achieved ceiling accuracy for the standard and deviant stimuli (SI group = 95.05%, NSI group = 94.55%), and t-test showed that there was no difference in accuracy between the two groups (*t* = 0.30, *p* = 0.762). The ANOVA of the reaction times (RTs) with stimuli type (deviant, standard) and group (SI, NSI) showed an effect of stimuli type [*F*(1, 42) = 328.35, *p* < 0.001, η*^2^p* = 0.887], the RTs for deviant stimuli (569.44 ± 6.30) were significantly longer than that of standard stimuli (489.93 ± 5.78); and an effect of group [*F*(1, 42) = 8.84, *p* = 0.005, η*^2^p* = 0.174], the RTs for SI group (546.42 ± 6.79) were significantly longer than that of NSI group (512.95 ± 8.98). The ANOVA with valence (happy, angry) and group (SI, NSI) showed an effect of group [*F*(1, 42) = 7.32, *p* = 0.010, η*^2^p* = 0.148], the RTs for SI group (586.48 ± 7.59) was significantly longer than that of NSI group (552.40 ± 10.05); and an interaction effect between valence and group [*F*(1, 42) = 7.01, *p* = 0.011, η*^2^p* = 0.143]. The simple-effect analyses of two-way interaction showed a significant group effect in valences [*F*(1, 42) = 10.06, *p* = 0.003, η*^2^p* = 0.193], with longer RTs for SI group (588.41 ± 44.21) than for NSI group (545.05 ± 42.55) in happy valence; and the valence effect was significant in group [*F*(1, 42) = 6.91, *p* = 0.012, η*^2^p* = 0.141], with longer RTs for angry valence (559.75 ± 9.93) than for happy valence (545.05 ± 10.91) in NSI group. It can be seen that participants showed significant reaction time delay under the experimental conditions due to the need of reaction inhibition.

**TABLE 1:** Averaged reaction times (RTs) and standard errors (SE) for each of the conditions in both groups (ms).

	**SI**	**NSI**		

**Stimuli type**	**M ± SD**	**M ± SD**	***F***	***p***
Standard	506.366.97	473.509.21	8.84	0.005
Deviation	586.487.59	552.4010.05		
**Emotion**				
Happy	588.4144.21	545.0542.55	7.32	0.010
Angry	584.5442.58	559.7533.94		

*SI, risky-suicidal ideation; NSI, non-suicidal ideation.*

### ERP Results

#### Test the Effect of BIC (Original Waveform)

The repeated ANOVA of the average amplitude of 280–360 ms interval with stimuli type (deviation, standard), electrode sites (frontal sites: F1, Fz, F2; frontal-central sites: FC1, FCz, FC2; central sites: C1, Cz, C2; central-parietal sites: CP1, CPz, CP2; parietal sites: P1, Pz, P2), and group (SI, NSI) was conducted. The results showed an effect of stimuli type [*F*(1, 42) = 5.01, *p* = 0.031, η*^2^p* = 0.107], the average amplitude for deviant stimuli (5.32 ± 0.84) was significantly larger than that of standard stimuli (4.43 ± 0.73); an effect of electrode sites [*F*(2, 80) = 75.26, *p* < 0.001, η*^2^p* = 0.642], largest N2 amplitudes were recorded at frontal electrode sites, and all anterior sites displayed larger N2 than posterior sites, in which Fz site recorded the largest N2 amplitude and P2 site recorded the smallest; an effect of group [*F*(1, 42) = 11.53, *p* = 0.002, η*^2^p* = 0.215], the average amplitude of SI group (2.30 ± 0.92) was significantly more negative than that of NSI group (7.46 ± 1.21); as well as an interaction effect between stimuli type and electrode sites [*F*(2, 74) = 13.30, *p* < 0.001, η*^2^p* = 0.241] showed the standard stimuli elicited a significantly larger negative component than deviant stimuli especially in frontal sites (as seen in Figure 3).

**FIGURE 4:**
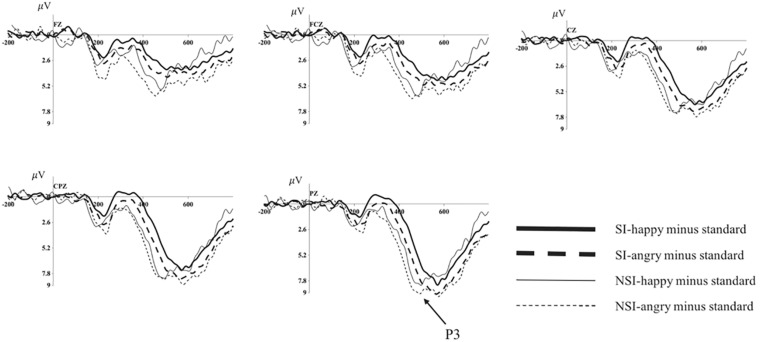
Average of difference ERPs at Fz, FCz, Cz, CPz, and Pz for angry and happy in SI group and NSI group. SI, risky-suicidal ideation; NSI, non-suicidal ideation.

The repeated ANOVA of the average amplitude of 450–510 ms interval with stimuli type (deviation, standard), electrode sites (frontal sites: F1, Fz, F2; frontal-central sites: FC1, FCz, FC2; central sites: C1, Cz, C2; central-parietal sites: CP1, CPz, CP2; parietal sites: P1, Pz, P2), and group (SI, NSI) was conducted. The results showed an effect of stimuli type [*F*(1, 42) = 114.24, *p* < 0.001, η*^2^p* = 0.731], the average amplitude for deviant stimuli (12.23 ± 0.75) was significantly larger than that of standard stimuli (6.40 ± 0.55); an effect of electrode sites [*F*(2, 75) = 43.54, *p* < 0.001, η*^2^p* = 0.509], largest P3 amplitudes were recorded at parietal electrode sites, and all posterior sites displayed larger P3 than anterior sites, in which Pz site recorded the largest P3 amplitude and F1 site recorded the smallest; an effect of group [*F*(1, 42) = 5.07, *p* = 0.03, η*^2^p* = 0.108], the average amplitude of NSI group (10.68 ± 0.95) was significantly more positive than that of SI group (8.01 ± 0.72); as well as an interaction effect between stimuli type and electrode sites [*F*(2, 89) = 29.06, *p* < 0.001, η*^2^p* = 0.409] showed the deviant stimuli elicited larger positive component than standard stimuli especially in the parietal sites, which exhibits significant P3 activity in the difference wave (as seen in Figures 3, 4). In addition, there was a marginal significant interaction effect of stimuli type and group [*F*(1, 42) = 3.80, *p* = 0.058, η*^2^p* = 0.083]. The simple-effect analyses of two-way interaction showed a group effect in stimuli types [*F*(1, 42) = 6.30, *p* = 0.02, η*^2^p* = 0.131], with larger amplitude for NSI group (14.16 ± 1.19) than for SI group (10.41 ± 0.90) in deviant stimuli, and there was no difference between the two groups in standard stimuli. It can be seen that the deviant stimuli elicited a significant behavioral inhibition effect under the experimental conditions, which was concentrated in the parietal sites difference P3 activity.

#### Differences of BIC Processes in Group of SI and NSI (Deviation-Standard Difference Wave Analysis)

The repeated ANOVA of the difference wave of P3 with valence (angry, happy), electrode sites (frontal sites: F1, Fz, F2; frontal-central sites: FC1, FCz, FC2; central sites: C1, Cz, C2; central-parietal sites: CP1, CPz, CP2; parietal sites: P1, Pz, P2), and group (SI, NSI) was conducted. The results showed an effect of electrode sites [*F*(2, 89) = 29.64, *p* < 0.001, η*^2^p* = 0.414], largest P3 amplitudes were recorded at parietal electrode sites, and all posterior sites displayed larger P3 than anterior sites, in which Pz site recorded the largest P3 amplitude and F1 site recorded the smallest; and an effect of group [*F*(1, 42) = 4.28, *p* = 0.045, η*^2^p* = 0.092], the difference wave of NSI group (7.07 ± 0.87) was significantly larger that of SI group (4.80 ± 0.67) regardless of the electrode sites and valence. In addition, there was a marginal significant effect of valence [*F*(1, 42) = 3.87, *p* = 0.056, η*^2^p* = 0.084], the difference wave was larger in angry valence (6.40 ± 0.64) than that of happy valence (5.47 ± 0.55). The analysis of the peak latency showed an effect of electrode sites [*F*(6, 232) = 4.42, *p* < 0.001, η*^2^p* = 0.095], longest P3 latencies were recorded at parietal electrode sites, and all posterior sites displayed longer P3 latencies than anterior sites, in which P1 site recorded the longest P3 latency and Fz site recorded the shortest; and an effect of group [*F*(1, 42) = 8.98, *p* = 0.005, η*^2^p* = 0.176], the latency of SI group (489.98 ± 1.81) was significantly longer than that of NSI group (480.98 ± 2.40) (as seen in Figure 4).

#### The Explorations on the Effect of Emotions in the BIC of SI and NSI

Since the marginal significant effect of valence in the analysis of difference waveform, we analyzed the differences of BIC in happy and anger faces in SI and NSI group respectively to clarify exactly which group has significant emotional effects. The ANOVA of difference wave of SI group or NSI group with valence (angry, happy) and electrode sites (frontal sites: F1, Fz, F2; frontal-central sites: FC1, FCz, FC2; central sites: C1, Cz, C2; central-parietal sites: CP1, CPz, CP2; parietal sites: P1, Pz, P2) was conducted. In SI group, the analysis showed an effect of valence in P3 amplitude [*F*(1, 27) = 8.36, *p* = 0.007, η*^2^p* = 0.237], the amplitude was more positive in angry valence (5.46 ± 0.62) than that of happy valence (4.14 ± 0.60); and an effect of electrode sites in P3 amplitude [*F*(2, 61) = 21.11, *p* < 0.001, η*^2^p* = 0.439], largest P3 amplitudes were recorded at parietal electrode sites, and all posterior sites displayed larger P3 than anterior sites, in which Pz site recorded the largest P3 amplitude and F1 site recorded the smallest; as well as an effect of electrode sites in P3 latency [*F*(6, 152) = 2.65, *p* = 0.02, η*^2^p* = 0.089], longest P3 latencies were recorded at parietal electrode sites, and all posterior sites displayed longer P3 latencies than anterior sites, in which P1 site recorded the longest P3 latency and F1 site recorded the shortest. In NSI group, the analysis showed an effect of electrode sites in P3 amplitude [*F*(2, 26) = 10.20, *p* = 0.001, η*^2^p* = 0.405], largest P3 amplitudes were recorded at parietal electrode sites, and all posterior sites displayed larger P3 than anterior sites, in which Pz site recorded the largest P3 amplitude and F1 site recorded the smallest.

## Discussion

Employing a two-choice oddball paradigm, the present study sheds light on two issues from a neural perspective: one is the difference in BIC between SI and NSI groups; the other is how angry and happy emotions impact BIC in the two groups. The results suggest that individuals without SI have better BIC, and individuals with SI need a more effortful BIC to complete the suppression of happy emotions. Different from most previous studies that used self-report scales to measure impulsivity, the present study used laboratory measurements to assess and compare BIC between SI and NSI groups, thereby providing intuitive evidence for the underlying mechanism of the effect of impulsivity on SI. Moreover, the present study highlights the effect of different emotions on the BIC of SI from an electrophysiological perspective, which provides an experimental basis for the study of the risk factors for SI.

The analysis of the original waveform reflects the validity of the two-choice oddball paradigm in measuring BIC. Given that response conflicts should be high when a low-frequency response must be made in the context of producing stereotyped or habitual responses (Braver et al., 2001; Nieuwenhuis et al., 2003), the dominant response of standard stimuli (high-frequency response) needs to be suppressed to ensure a correct response to deviant stimuli (low-frequency response), which requires more effort. Thus, a significant difference in P3 amplitudes was induced by the deviant and standard stimuli, thus indicating the effect of BIC, which was consistent with previous studies (Yuan et al., 2007, Yuan et al., 2008b). The N2 amplitude of standard stimuli in the present study was larger than that of deviant stimuli, which was contrary to previous studies (Yuan et al., 2008b; Xin et al., 2010). There may be two reasons for this result: one is that arousal in response to facial stimuli is weaker than arousal in response to evocative pictures, as the former may not be salient enough to induce an alert response of attention toward novel stimuli in the early stage of attention (Britton et al., 2006); the other is that the deviant stimuli in this experiment were repetitive and repeated many times (e.g., each emotional face of a model was repeated nine times), which reduced the unpredictability and made participants familiar with the stimuli, leading to the lack of an orientation response to novelty stimuli (Daffner et al., 2000).

The difference waveform is a sensitive index for measuring BIC in the two-choice oddball paradigm. The P3 component is considered an indicator of the processing of BIC (Donkers and Van Boxtel, 2004; Albert et al., 2010), and the amplitude induced by BIC is significantly larger than that induced by uncontrolled conditions (Yu et al., 2009). The P3 amplitude elicited in the NSI group was significantly larger than that of the SI group in the present study, indicating that the BIC of the NSI group was better than that of individuals with SI. According to the theory of Eysenck (1993), high impulsivity demonstrates reduced cognitive performance, which is reflected in a reduced P3 amplitude, and the P3 amplitude with cognitive control is smaller than that without cognitive control (Chen et al., 2008). The results suggest that individuals with SI need to consume more cognitive control to eliminate the interference of unrelated information to ensure the effective completion of the BIC process. Similarly, the P3 latency of individuals with SI was longer than that of the NSI group, which also indicates that the SI group takes longer to complete the BIC process. As a result of the deficit of cognitive control in suicidal ideation (Richard-Devantoy et al., 2013), this suggests that cognitive control may be a potential mechanism between suicidal ideation and impulsivity. Specifically, the ability of cognitive control is impaired when individuals develop SI, which further leads them to have difficulties orchestrating threatening thoughts and actions, thus increasing the risk of suicide. Based on the results of this study, identifying the internal mechanism of the high correlation between impulsivity and suicidal ideation is critical for the prevention of suicide.

Regarding the effect of emotion on BIC, the present study found that the P3 amplitude in response to happy faces was smaller than that in response to angry faces in the SI group, whereas the NSI group had no significant differences in BIC between the two emotions. In light of the P3 amplitude decreases in the condition of cognitive control (Chen et al., 2008), individuals with SI require more cognitive control to eliminate the interference of unrelated information to complete the process of inhibitory control under the condition of happy faces. In contrast to a previous study of healthy individuals showing that BIC was weaker in response to negative emotions (Yuan et al., 2007, 2012), the current study found that BIC decreased in response to positive emotions among the SI group. The Broaden-and-Build Model of Positive Emotions suggests that positive emotions significantly expand the scope and increase the flexibility of attention (Fredrickson, 1998; Schmitz et al., 2009), which makes individuals pay attention to the integrity of information and ignore the details of the information, thus making them unable to concentrate on the completion of the control task and hindering their BIC. Moreover, the reduced P3 amplitude that reflects impulsivity is usually interpreted as indicating a reduction in attentional resources that are available for information processing because these resources are not allocated effectively or because of decreased physiological arousal (Russo et al., 2008). In this way, the reason for this result may be that individuals with SI pay less attention to positive stimuli (Jollant et al., 2008), and they require more effort to exhibit BIC in response to happy (vs. angry) faces. Individuals with SI have acceptable BIC in response to angry faces, which could be explained by the model of cognitive control of emotion (MCCE). MCCE suggests that there are four steps involved in generating emotional responses (Ochsner et al., 2012): perceiving stimuli in the environment, deploying attention to these stimuli, appraising the significance of stimuli, and responding to the stimuli, including automatic physiological responses. P3 component shows differences in the process of appraising stimuli, which represent the cognitive evaluation of the meaning of stimuli (Ito et al., 1998; Huang and Luo, 2006). Specifically, angry faces indicate more threatening information, and individuals tend to employ more attention to these faces and evaluate it as negative information, which would recruit more physiological and psychological resources and elicit a higher P3 amplitude. This result shows that different emotions have an effect on the BIC of the SI group but not the NSI group, which is helpful for understanding the relationship between impulsivity and SI from the role of emotion.

In addition, the present study points to two important contributions. First, we clarified the neural mechanism of BIC. Previous studies on BIC that employed fMRI technology found that the function of BIC associated with the fronto-basal ganglia networks, including the ventromedial prefrontal cortex, the prefrontal cortex of the central anterior, the orbitofrontal cortex, and the anterior cingulate cortices (ACC), plays an important role (Li et al., 2006; Aron, 2007; Dillon and Pizzagalli, 2008). Consistent with the results of brain imaging studies, the P3 component found in the present study was closely related to the inhibitory control of emotion and was mainly distributed in the central-parietal region, and the sources of P3 component were mainly in the ACC and lateral orbitofrontal cortex, which were revealed to be the major brain functional regions of emotional inhibitory control (Albert et al., 2010). Moreover, the present study provided more empirical evidence for the theory of suicidal ideation, leading the “emotion-impulsivity framework” of suicidal ideation to an ERP experiment. Based on previous studies only involving the impact of one of impulsivity or emotion on suicidal ideation (Albanese et al., 2019; Sarkisian et al., 2019; Wang et al., 2019), the ERP activities related to impulsivity–emotion interaction and suicidal ideation were explored in this study. The present findings could lay the foundation for further research on the etiological pathways of suicidal ideation employing fMRI.

Finally, some limitations of the present study should be outlined. First, the proportion of females was larger than that of males in the present study. Gender differences in BIC were found in a previous study (Fillmore and Weafer, 2004), and the advantage of females in BIC has been proven (Yuan et al., 2008a). Furthermore, gender differences in ERP components during response inhibition have been highlighted by other authors (Li et al., 2009; Huster et al., 2011). It is possible that the unbalanced proportion of gender might affect the results of this study. Second, the number of participants in the SI and NSI groups was unbalanced in the present study. Individuals with SI were more likely to refuse to participate in the experiment when they were recruited, which led us to find more SI participants and ultimately led to the imbalance of groups under strict grouping criteria. This might reduce the generalizability of the research results to some extent. Third, the different types of facial materials were not conspicuous enough and were repeated many times, which may not have attracted enough attention in the early stage of material presentation. This might account for the deficit of the N2 effect in the present study, which indicates conflict monitoring. Despite these limitations, we believe the present study encourages the use of psychophysiological indexes such as the P3 amplitude to measure and compare BIC between SI and NSI groups. The present study also provides a deeper understanding of the intriguing relationship among impulsivity, emotion, and SI.

## Data Availability Statement

The datasets generated for this study are available on request to the corresponding author.

## Ethics Statement

The studies involving human participants were reviewed and approved by the Ethics Committee of Tianjin Normal University. The participants provided written informed consent to participate in this study.

## Author Contributions

CW who has contributed to the study equally as LL is the co-first author of the manuscript. LL and CW designated the experiment. CW, JM, YL, and TL conducted the experiment. LL, CW, YJ, and XW analyzed the data and wrote the manuscript. LL and XW critically reviewed the manuscript. XB provided suggestions in reply to the review comments. All authors gave final approval of the version to be submitted.

## Conflict of Interest

The authors declare that the research was conducted in the absence of any commercial or financial relationships that could be construed as a potential conflict of interest.
